# COVID-19-related school closing aggravate obesity and glucose intolerance in pediatric patients with obesity

**DOI:** 10.1038/s41598-021-84766-w

**Published:** 2021-03-09

**Authors:** Eun Sil Kim, Yiyoung Kwon, Yon Ho Choe, Mi Jin Kim

**Affiliations:** Department of Pediatrics, Samsung Medical Center, Sungkyunkwan University School of Medicine, Seoul, Korea

**Keywords:** Endocrinology, Medical research

## Abstract

It is important to pay attention to the indirect effects of the social distancing implemented to prevent the spread of coronavirus disease 2019 (COVID-19) pandemic on children and adolescent health. The aim of the present study was to explore impacts of a reduction in physical activity caused by COVID-19 outbreak in pediatric patients diagnosed with obesity. This study conducted between pre-school closing and school closing period and 90 patients aged between 6- and 18-year-old were included. Comparing the variables between pre-school closing period and school closing period in patients suffering from obesity revealed significant differences in variables related to metabolism such as body weight z-score, body mass index z-score, liver enzymes and lipid profile. We further evaluated the metabolic factors related to obesity. When comparing patients with or without nonalcoholic fatty liver disease (NAFLD), only hemoglobin A1c (HbA1c) was the only difference between the two time points (*p* < 0.05). We found that reduced physical activity due to school closing during COVID-19 pandemic exacerbated obesity among children and adolescents and negatively affects the HbA1C increase in NAFLD patients compared to non-NAFLD patients.

## Introduction

The current coronavirus disease 2019 (COVID-19) pandemic has led governments of affected countries to impose strict confinement rules, that is social distancing, on their citizens including children and adolescents. All citizens in the Republic of Korea have been asked to restrict their social interactions and live under home-confinement for several months since February 2020. School classes have been replaced by online classes according to the social distancing and stay-at-home orders. The result of these policies has led to social, economic, psychological, and health implications.

It is important to pay attention to the indirect effects of COVID-19 pandemic on children and adolescent health caused by compulsory sedentary lifestyle. It is reported that there were decreased physical activity and weight gain in 56.7% of school-aged children and adolescents in the Republic of Korea during COVID-19 pandemic^1^. The school closing due to COVID-19 pandemic may reduce physical activity and aggravate obesity and increase the risk of other metabolic diseases in pediatric population. However, there are scarce data on the impact of COVID-19 related school closing on pediatric health.

Obesity occurs as the result of interactions between genetic and environmental factors^2–4^; however, school and out-of-school environments related to decreased physical activity are factors that may contribute to pediatric obesity in school-aged children^5–7^. Von Hippel et al. described that non-school environments contribute to excessive weight gain in childhood^5^, and Smith reported that children who have overweight or obesity tend to have more weight gain and increase in body mass index (BMI) compared to children who have normal weight during out-of-school period^7^. Other studies have also revealed metabolic impact of summer vacation or holiday on childhood obesity in terms of aggravating obesity since children have less opportunity for group exercise due to absence of physical education classes and school activities during vacation^6,8–12^.

There are well documented long-term effects of childhood obesity including asthma, type 2 diabetes, dyslipidemia, and hypertension^13–15^. Recent studies have emphasized that obesity can lead to nonalcoholic fatty liver disease (NAFLD) and metabolic syndrome and diabetes, which can persist into adulthood^16,17^. Therefore, efforts should be focused primarily on preventing, reducing and treating childhood and adolescent obesity.

The primary aim of the present study was to investigate change of laboratory results (e.g. liver enzymes, lipid profile, and hemoglobin A1c; HbA1c) according to the reduction in physical activity in pediatric patients with obesity during the period of school closing caused by COVID-19 outbreak. The secondary aim of this study was to evaluate the indirect metabolic effects of sedentary life style caused by COVID-19 outbreak in patients diagnosed with NAFLD among pediatric patients with obesity.

## Materials and methods

### Patients and data collection

**Figure 1:**
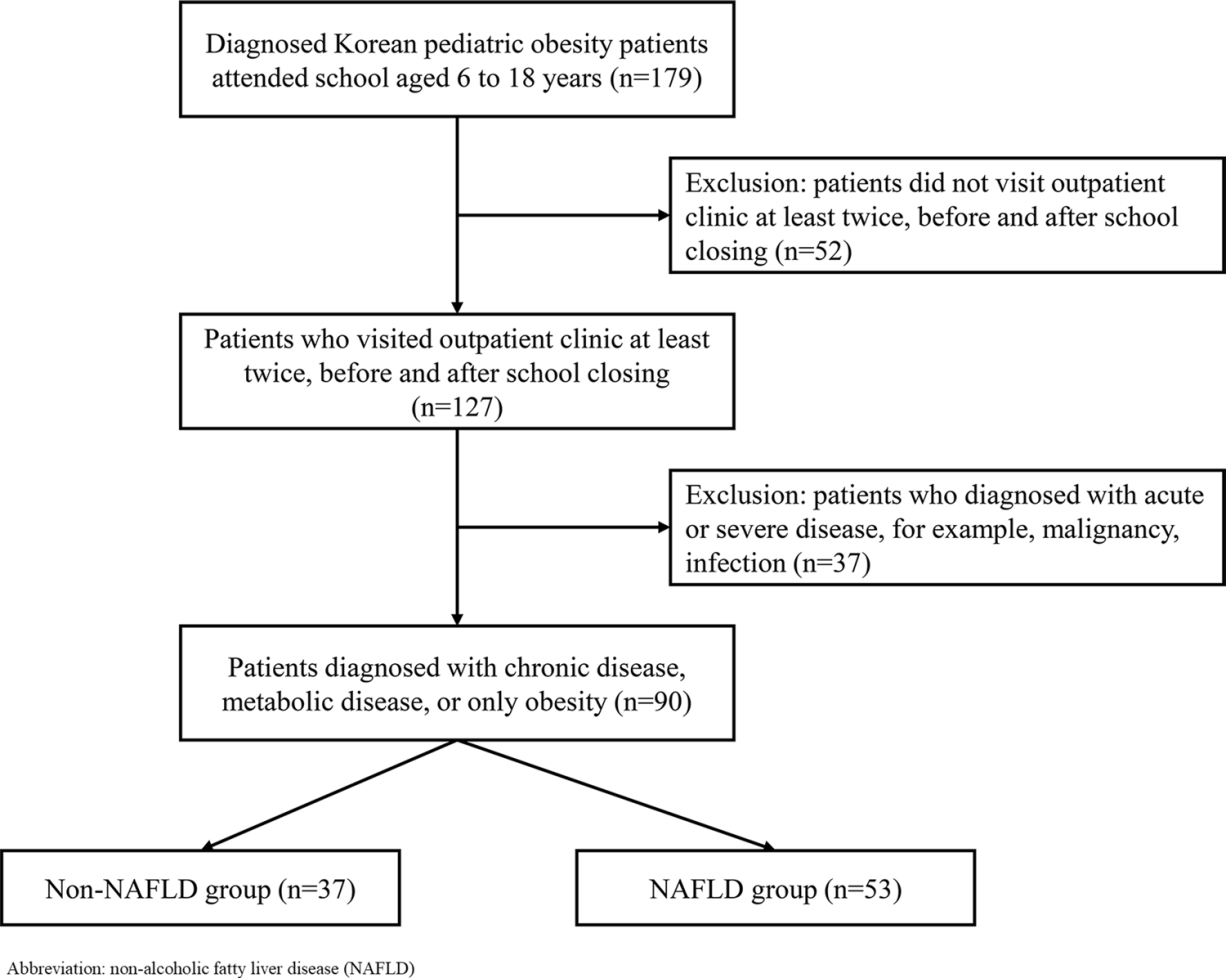
Flow diagram of the study.

This study was a retrospective observational study conducted at the Department of Pediatrics of Samsung Medical Center between December 2019 and May 2020. The subjects were pediatric patients with obesity who attended school between aged 6 and 18, and visited the outpatient clinic at least twice before and during school closing period due to COVID-19 outbreak. During the study period, subjects were all at their homes because their school program was suspended due to COVID-19 outbreak. It was assumed that pre-school closing was from December 2019 to February 2020 and school closing was from March 2020 to May 2020. Patients were identified through a search of our electronic medical records system. In the database search, we identified 179 school-aged patients diagnosed with obesity and the number of patients visited outpatient clinic at least twice before and after social distancing was 127. Then, 37 patients were excluded because of alternative etiologies for elevated liver enzymes, and 90 patients were eligible for analysis finally (Fig. 1).

Demographic and clinical data including sex, age, body weight, height, and BMI were collected before and after school closing. BMI was calculated as weight/height^2^ (kg/m^2^). Z-scores for body weight and BMI were calculated based on the 2017 Korean National Growth Charts for children and adolescents^18^. Data at each visit to the outpatient clinic were collected retrospectively from electronic charts and laboratory results, including aspartate aminotransferase (AST), alanine aminotransferase (ALT), fasting glucose, uric acid, cholesterol, triglyceride, high-density lipoprotein (HDL), low-density lipoprotein (LDL), and HbA1c. We compared data between pre-school closing and during school closing and also according to the presence of NAFLD.

### Definitions

Obesity was defined as a BMI above the 95th percentile in children and adolescents in accordance with the Committee on Pediatric Obesity of the Korean Society of Pediatric Gastroenterology Hepatology and Nutrition^19^. NAFLD was diagnosed by excluding alternative etiologies for elevated ALT and/or hepatic steatosis, such as medication (amiodarone, glucocorticoids, L-asparaginase, valproic acid) and the presence of co-existing chronic liver disease including Wilson’s disease, type 1 diabetes mellitus, hepatitis B, and hepatitis C^20^. It is important to note that not all pediatric patients with obesity and chronic elevated liver enzymes are classified as NAFLD. The gold standard for establishing a NAFLD diagnosis is liver biopsy; however, there are the serious limitations to liver biopsy in pediatric patients in the real-world setting. Therefore, NAFLD was diagnosed based on three conditions; elevated ALT (> 50U/L in boys, > 44 U/L in girls)^21^, increased brightness of the liver parenchyma compared to the kidney in liver ultrasonography conducted by one pediatric radiology specialist^20^, and excluding other etiologies for hepatic steatosis as described above^22^.

### Statistical analysis

Comparative data for continuous variables are reported as the median with interquartile range (IQR) or mean with standard deviation and for categorical variables with frequency and percentages. The analysis of variance (ANOVA) test was used to compare continuous variables while the chi-square test was used for discrete variables. We used paired *t*-tests to evaluate the significance of changes from pre-school closing to school closing period. In addition, we tested the significance of differences between patients with and without NAFLD in response to changes using independent two-sample *t*-tests. Statistical significance was defined as *p* < 0.05. Statistical analyses were performed using Rex (Version 3.0.3, RexSoft Inc., Seoul, Korea).

### Ethics declarations

This study was approved by the Institutional Review Board of the Samsung Medical Center and was conducted in accordance with the Declaration of Helsinki^23^. All patients and parents and/or legal guardian of subjects who are under 18 provided written informed consent. We confirmed that all methods were performed in accordance with the approved guidelines and regulations. We reported and presented data according to the STROBE statement.

## Results

### Baseline characteristics

During the period from December 2019 to May 2020, a total of 90 pediatric patients with obesity visited the clinic at least two times between the pre-school closing and during school closing period. Table 1 shows descriptive characteristics of subjects at baseline. The mean age of patients at pre-school closing was 12.2 ± 3.4 years and 70 patients (77.8%) were male. The median interval between first visit of outpatient clinic (pre-school closing) and second visit (during school closing) was 4.3 months. Mean body weight z-score was 2.0 and mean BMI z-score was 1.9. At baseline, 53 (58.9%) subjects had NAFLD, 10 (11.1%) had type 2 diabetes mellitus, and 14 (15.6%) had dyslipidemia. In addition, statin usage was observed in 13 subjects (14.4%), metformin usage was in 10 subjects (11.1%), and insulin usage was in 1 subject (1.1%), which was maintained during the study period. Other baseline demographics and clinical characteristics, as well as data collected at diagnosis, are summarized in Table 1.

**Table 1:** Baseline characteristics of pediatric patients with obesity.

	Total (*n* = 90)
Age (years)	12.2 ± 3.4
Male, *n* (%)	70 (77.8)
Female, *n* (%)	20 (22.2)
Interval between 1st and 2nd outpatient clinic visit (months)	4.3 ± 1.8
Body weight (kg)	67.2 ± 23.8
Body weight z-score	2.0 ± 0.8
BMI (kg/m^2^)	26.7 ± 4.6
BMI *z*-score	1.9 ± 0.5
Mean arterial pressure	86.5 ± 9.1
**Co-existing diseases, *n * (%)**	
NAFLD	53 (58.9)
Type 2 diabetes mellitus	10 (11.1)
Dyslipidemia	14 (15.6)
Polycystic ovarian syndrome	2 (10)
**Medication, *n* (%)**	
Statins	13 (14.4)
Metformin	10 (11.1)
Insulin	1 (1.1)
AST (U/L)	35.2 ± 29.0
ALT (U/L)	53.0 ± 32.8
Fasting glucose (mg/dl)	160.3 ± 33.4
Uric acid (mg/dl)	6.4 ± 1.9
Cholesterol (mg/dl)	160.3 ± 33.4
Triglyceride (mg/dl)	126.7 ± 70.0
HDL (mg/dl)	46.9 ± 11.1
LDL (mg/dl)	101.5 ± 31.0
HbA1c (%)	6.0 ± 1.3

BMI body mass index, NAFLD non-alcoholic fatty liver disease, AST aspartate transferase, ALT alanine transferase, HDL high-density lipoprotein, LDL low-density lipoprotein, HbA1c hemoglobin A1c.

### Comparison of variables between pre-school closing and during school closing in patients with obesity

**Figure 2:**
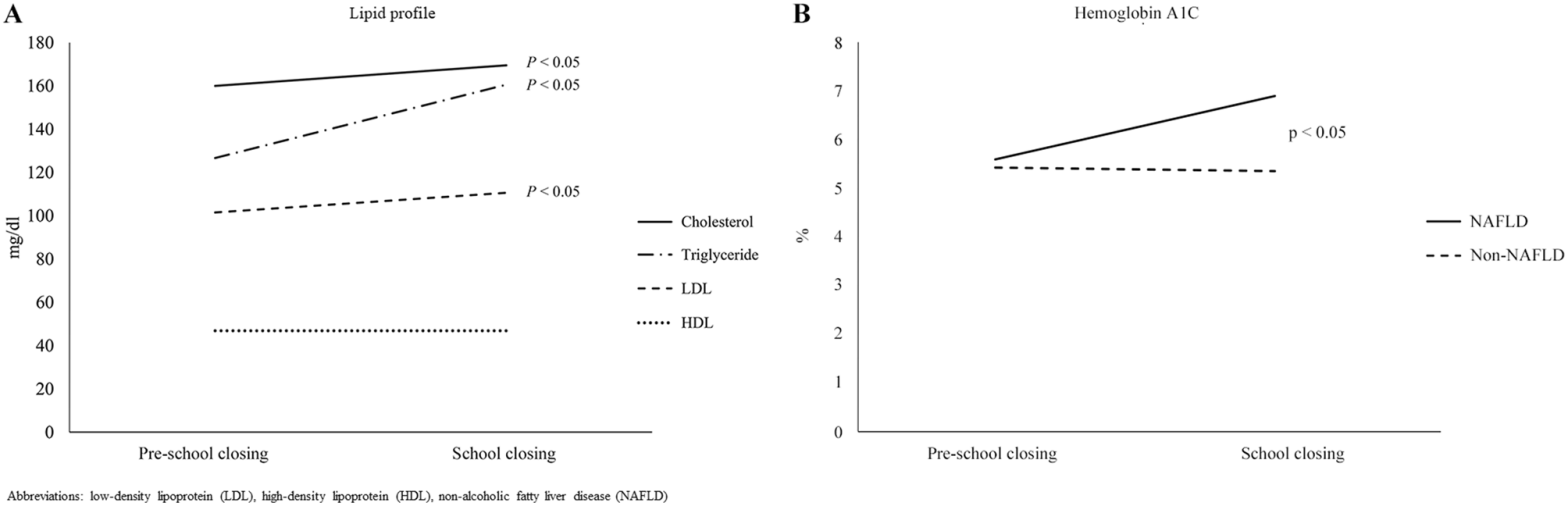
Comparison laboratory results between the pre-school closing and during school closing. (**A**) Lipid profile, (**B**) HbA1C between with and without NAFLD group.

We investigated the changes in growth in weight, height, and BMI and other laboratory results associated with metabolism between the pre-school closing and during school closing. Comparing the variables between pre-school closing period and school closing period in patients suffering from obesity revealed significant differences in variables related to metabolism such as body weight (67.2 ± 23.8 vs. 71.1 ± 24.2, *p* < 0.001), body weight z-score (2.0 ± 0.8 vs. 2.2 ± 0.7, *p* < 0.001), BMI (26.7 ± 4.6 vs. 27.7 ± 4.6, *p* < 0.001), BMI z-score (1.9 ± 0.5 vs. 2.0 ± 0.4, *p* < 0.001), AST (35.2 ± 29.0 vs. 42.7 ± 33.8, *p* = 0.0085), ALT (53.0 ± 32.8 vs. 74.7 ± 41.8, *p* < 0.001). Also, lipid profile such as cholesterol (160.3 ± 33.4 vs. 169.5 ± 36.4, *p* < 0.001), triglycerides (126.7 ± 70.0 vs. 160.6 ± 94.0, *p* < 0.001), and LDL (101.5 ± 31.0 vs. 110.6 ± 33.7, *p* = 0.0016) increased remarkably during school closing compared to pre-school closing. (Fig. 2A). There were no statistically significant differences between the two time points in mean blood pressure (MBP), uric acid, HDL, and HbA1c. Detailed comparison results are presented in Table 2.

**Table 2:** Comparison of variables between pre-school closing and during school closing in patients with obesity.

	Pre-school closing	School closing	*p* value
Body weight (kg)	67.2 ± 23.8	71.1 ± 24.2	< 0.001
Body weight z-score	2.0 ± 0.8	2.2 ± 0.7	< 0.001
BMI (kg/m^2^)	26.7 ± 4.6	27.7 ± 4.6	< 0.001
BMI z-score	1.9 ± 0.5	2.0 ± 0.4	< 0.001
Mean arterial pressure (mmHg)	86.5 ± 9.1	89.2 ± 9.7	0.1073
AST (U/L)	35.2 ± 29.0	42.7 ± 33.8	0.0085
ALT (U/L)	53.0 ± 32.8	74.7 ± 41.8	< 0.001
Fasting glucose (mg/dl)	160.3 ± 33.4	169.5 ± 36.4	< 0.001
Uric acid (mg/dl)	6.4 ± 1.9	7.3 ± 10.6	0.4684
Cholesterol (mg/dl)	160.3 ± 33.4	169.5 ± 36.4	< 0.001
Triglyceride (mg/dl)	126.7 ± 70.0	160.6 ± 94.0	< 0.001
HDL (mg/dl)	46.9 ± 11.1	46.9 ± 10.7	0.9715
LDL (mg/dl)	101.5 ± 31.0	110.6 ± 33.7	0.0016
HbA1c (%)	6.0 ± 1.3	7.3 ± 6.0	0.1737

BMI body mass index, NAFLD non-alcoholic fatty liver disease, AST aspartate transferase, ALT alanine transferase, HDL high-density lipoprotein, LDL low-density lipoprotein, HbA1c hemoglobin A1c.

### Comparison of variable differences between patients with and without NAFLD

In this retrospective analysis of 90 pediatric patients with obesity, 53 patients (58.9%) had NAFLD. At the pre-school closing period, the NAFLD group showed higher AST (30 vs. 24, *p* <0.001), ALT (54 vs. 17, *p* <0.001), serum uric acid (4.4 vs. 4.0, *p* <0.001), and HbA1c (5.6 vs. 5.4, *p* <0.001), and lower LDL (95.2 vs. 109.8, *p* <0.05) relative to the non-NAFLD group. During the school closing period, the NAFLD group showed higher AST (44 vs. 23, *p* <0.001), ALT (70 vs. 22, *p* <0.001), HbA1c (6.9 vs. 5.4, *p* <0.001), and MBP (98 vs. 86.7, *p* <0.05) compared to the non-NAFLD group (Table 3). Other detailed comparison results are presented in Table 3.

We also compared incremental change (delta value) in each variable at the two time points of pre-school closing and during school closing according to the presence of NAFLD. The delta values were calculated by subtracting the value of the pre-school closing from the value of the school closing period. Comparison of the delta values between patients with or without NAFLD revealed significant difference in only HbA1c levels at the two time points (0.35 vs. 0.05, *p* < 0.05) except for liver enzymes. There were no other significant differences between the two time points in the NAFLD and non-NAFLD groups (Table 4 and Fig. 2B).

**Table 3:** Comparison of variables according to the presence of NAFLD between pre-school closing and during school closing period.

Variables	Pre-school closing			School closing		
	NAFLD (*n* = 53)	Non-NAFLD (*n* = 37)	*p* value	NAFLD (*n* = 53)	Non-NAFLD (*n* = 37)	*p* value
Age (years)	12.9 (11.5, 14.6)	10.3 (7.6, 12.7)	< 0.001			
Male, *n* (%)	45 (84.9)	25 (67.6)	0.0912			
Female, *n* (%)	8 (15.1)	12 (32.4)	0.0912			
Body weight z-score	2.10 (1.74, 2.54)	1.95 (1.65, 2.51)	0.6025	2.17 (1.89, 2.77)	2.14 (1.86, 2.57)	0.9444
BMI z-score	1.96 (1.61, 2.06)	1.97 (1.56, 2.28)	0.6053	2.02 (1.76, 2.16)	1.99 (1.75, 2.30)	0.5576
Mean arterial pressure (mmHg)	86.3 (81.2, 89.3)	85.7 (81.3, 91.7)	0.9839	98.0 (90.3, 101.3)	86.7 (82.6, 89.3)	0.0027
AST (U/L)	30 (23, 41)	24 (20. 27)	< 0.001	44 (31, 59)	23 (21, 25)	< 0.001
ALT (U/L)	54 (35, 71)	17 (14, 30)	< 0.001	70 (53, 117)	22 (17, 26)	< 0.001
Fasting glucose (mg/dl)	97 (90, 104)	97 (91, 104)	0.8334	101 (93, 116)	99 (92, 114)	0.34
Uric acid (mg/dl)	5.5 (6.0, 7.2)	4.9 (3.9, 5.0)	< 0.001	6.9 (5.7, 7.7)	5.7 (4.3, 6.8)	0.0021
Cholesterol (mg/dl)	151 (136, 171)	160 (144, 184)	0.1598	157 (142, 177)	170 (150, 210)	0.2488
Triglyceride (mg/dl)	118 (71, 138)	114 (92, 149)	0.8334	147 (85, 217)	150 (87, 232)	0.7604
HDL (mg/dl)	47.0 ± 10.4	49.7 ± 12.7	0.3027	45.1 ± 9.0	48.5 ± 13.7	0.2629
LDL (mg/dl)	95.2 ± 27.8	109.8 ± 29.6	0.0296	101.5 (89.0, 116.5)	105.5 (89.5, 133.75)	0.4744
HbA1c (%)	5.6 (5.4, 8.6)	5.4 (5.2, 5.6)	0.0029	6.9 (5.6, 8.9)	5.4 (5.3, 5.6)	< 0.001

BMI body mass index, NAFLD non-alcoholic fatty liver disease, AST aspartate transferase, ALT alanine transferase, HDL high-density lipoprotein, LDL low-density lipoprotein, HbA1c hemoglobin A1c.

**Table 4:** Comparison of difference in each variable at pre-school closing and during school closing according to the presence of NAFLD.

School closing—pre-school closing	Total (*n* = 90)	NAFLD group (*n* = 53)	Non-NAFLD group (*n* = 37)	*p* value
△ BMI z-score	0.06 (0, 0.12)	0.06 (0.03, 0.12)	0.06 (− 0.01, 0.12)	0.33
△ Body weight z-score	0.18 (0.1, 0.29)	0.18 (0.1, 0.25)	0.17 (0.09, 0.35)	0.95
△ MAP (mmHg)	4.33 (− 1.33, 7.33)	3.67 (− 1.5, 7.33)	5.33 (0.83, 11)	0.20
△ Cholesterol (mg/dl)	9.21 ± 18.06	7.3 ± 15.65	12.31 ± 21.33	0.28
△ AST (U/L)	4 (− 1.5, 14)	10 (0, 25)	− 0.5 (− 3.75, 3.75)	< 0.01
△ ALT (U/L)	8 (0, 35.5)	17 (3, 59)	2 (− 1, 7)	< 0.01
△ Fasting glucose (mg/dl)	2 (− 2, 11)	5 (− 0.5, 13.5)	0 (− 2.75, 4.75)	0.16
△ Uric acid (mg/dl)	0.35 (− 0.2, 0.7)	0.4 (0, 0.6)	0.2 (− 0.5, 0.8)	0.94
△ Triglyceride (mg/dl)	33.89 ± 61.15	26.39 ± 50.59	46.71 ± 75.37	0.25
△ HDL (mg/dl)	− 0.03 ± 6.87	− 0.45 ± 6.2	0.71 ± 8.01	0.54
△ LDL (mg/dl)	9 (− 3, 14)	9.5 (− 2.75, 13)	5 (− 3.25, 16.75)	0.76
△ HbA1c (%)	0.1 (0, 0.42)	0.35 (0.08, 1.55)	0.05 (0, 0.1)	< 0.05

delta, △ Subtracting the value of the pre-social distancing from the value of the social distancing period, BMI body mass index, NAFLD non-alcoholic fatty liver disease, MAP mean arterial pressure, AST aspartate transferase, ALT alanine transferase, HDL high-density lipoprotein, LDL low-density lipoprotein, HbA1c hemoglobin A1c.

## Discussion

To date, most studies have described the relationship between life style behaviors affected by COVID-19 and weight gain based on questionnaires, however there is scarce data about children and adolescents^24–29^. As far as we know, this is the first study to objectively investigate the indirect impact of COVID-19 pandemic on metabolic problems in pediatric patients with obesity using laboratory results.

In this study, the results show that COVID-19 pandemic has had a substantial negative impact on the health in pediatric patients with obesity and its comorbidities regardless of infection status of COVID-19. As a result of the current situation in which almost all people, even children and adolescents, are confined to their homes, physical activity drastically declined while dietary habits remained unchanged or failed to offset physical inactivity. We show that reduced physical activity caused by school closing in COVID-19 pandemic era could lead to a rapid decline in metabolic homeostasis. Notably, during the school closing period, there were remarkable increases in body weight, BMI, and laboratory results related to metabolic disease, such as AST, ALT, triglyceride, and LDL, which were statistically significant (*p* < 0.05).

In results according to the presence of NAFLD, NAFLD group had significantly higher MBP and HbA1c levels relative to non-NAFLD group during school closing period, which is consistent with other existing studies that reported children affected by obesity and NAFLD had a higher cardiovascular and metabolic risk, including hypertension^30,31^. In Table 2, HbA1c did not seem to change between before and during school closing; however, when subgroup analysis was performed with or without NAFLD, difference of HbA1c was significantly confirmed between the two time points (Table 3). Furthermore, in accordance with previous studies, children with NAFLD were more susceptible to increases in HbA1c than non-NAFLD pediatric patients caused by reduced physical activity during COVID-19 pandemic (*p* < 0.05)^16^. In other words, NAFLD may be an important predisposing factor in pediatric patients with obesity for the development of glucose intolerance, especially in environments with reduced physical activity^16,32^.

These observations not only recommend that patients with obesity do physical activities such as home training to the extent possible during school closing, but also emphasize that family members and pediatricians should pay attention to the lifestyle of pediatric patients with obesity. Although further studies in post-school closing are needed, aggravated obesity and other metabolic diseases during out-of-school circumstances may not be easily reversible and might contribute to excess adiposity and cardiometabolic or non-cardiometabolic comorbidities, which can persist into adulthood^2,19,33^.

Previous research has demonstrated that children gained significantly more body weight and showed increased BMI during out-of-school periods^5,6,8–12^. Some studies have suggested that individual weight gain during the holiday period was quite variable; however, this period is more important especially for those who have already been diagnosed with obesity^9,11^. Our findings are consistent with those of recent studies and indicate that out-of-school circumstances, such as school closing caused by COVID-19 outbreak, contribute to childhood and adolescent obesity and its comorbidities caused by low physical activity level.

The strength of this study is that body weight, BMI, and laboratory results associated with metabolic disease were objectively compared between two time points, pre-school closing and during school closing. On the other hand, our study has some limitations. First, as a retrospective study, it has certain limitations compared to a prospective design. However, all patients attended an outpatient clinic at least two times between pre-school closing and school closing periods and the extraction of objective clinical and biochemical results from the medical records was possible. Second, the lack of information about subject diet and exercise time is a limitation; however, such information is subjective, thus eliminating potential bias was possible in this study. Third, selection bias may have been introduced as patients who had not visited twice between the two time points were excluded from this study. Therefore, further well-designed prospective post-social distancing studies are required to address these limitations.

In conclusion, we found that reduced physical activity due to social distancing during COVID-19 pandemic exacerbated obesity among school-aged children and adolescents and negatively affects the HbA1C increase in NAFLD patients compared to non-NAFLD patients. These results lend support to the fact that physical activity is important in the prevention and treatment of obesity. Therefore, physicians should pay attention to life style modification as well as pharmacotherapy and surgery in pediatric patients with obesity. Moreover, physicians should carefully monitor the development of glucose intolerance in pediatric NAFLD patients during physical inactivity periods caused by school closing during COVID-19 pandemic.

## Data Availability

Please contact the corresponding author for data requests.
